# Draft Genome Sequence of Sodium-Independent Alkaliphilic *Microbacterium* sp. Strain TS-1

**DOI:** 10.1128/genomeA.01043-13

**Published:** 2013-12-19

**Authors:** Shun Fujinami, Kiyoko Takeda, Takefumi Onodera, Katsuya Satoh, Motohiko Sano, Issay Narumi, Masahiro Ito

**Affiliations:** Bio-Nano Electronics Research Centre, Toyo University, Kawagoe, Saitama, Japana; United Graduate School of Agricultural Science, Tokyo University of Agriculture and Technology, Fuchu, Tokyo, Japanb; Ion Beam Mutagenesis Research Group, Medical and Biotechnological Application Division, Quantum Beam Science Directorate, Japan Atomic Energy Agency, Takasaki, Gunma, Japanc; Graduate School of Life Sciences, Toyo University, Itakura-machi, Gunma, Japand; Faculty of Life Sciences, Toyo University, Itakura-machi, Gunma, Japane

## Abstract

Alkaliphilic *Microbacterium* sp. strain TS-1, newly isolated from the jumping spider, showed Na^+^-independent growth and motility. Here, we report the draft genome sequence of this bacterium, which may provide beneficial information for Na^+^-independent alkaline adaptation mechanisms.

## GENOME ANNOUNCEMENT

Alkaliphiles are microorganisms that can grow in alkaline environments, i.e., pH >9.0 (1). Alkaline adaptation mechanisms of Na^+^-dependent alkaliphilic bacillus species are among the most characterized in alkaliphiles (2). The typical alkaliphilic bacillus species has an Na^+^ cycle consisting of Na^+^ efflux and influx systems and shows sodium-dependent growth and motility (2, 3). On the other hand, some Na^+^-independent alkaliphiles have also been reported (4). There may be different alkaline adaptation mechanisms. Here, we report the draft genome sequence of *Microbacterium* sp. strain TS-1, which showed Na^+^-independent growth and motility. This bacterium was isolated from the jumping spider and appeared to be most closely related to *Microbacterium arborescens*, based on 16S rRNA gene sequence identity.

The draft genome sequence of *Microbacterium* sp. strain TS-1 was 3,396,165 bp in total length and comprised 3 large contigs (2,699,404 bp, 691,278 bp, and 5,483 bp). The sequence was obtained by using the Roche GS Junior and assembled by using the GS De Novo assembler v. 2.7. Automatic annotation was performed using the Microbial Genome Annotation Pipeline (5), which predicted a total of 3,156 protein-encoding genes. The names of products of predicted coding genes were revised manually for consistency. Forty-six tRNAs were predicted using the tRNAscan software (6).

The annotation of the draft genome sequence indicates that this bacterium has 3 sets of *mrp* genes, which encode multisubunit secondary cation/proton antiporter-3 family proteins. The Mrp complex acts as an Na^+^/H^+^ antiporter in alkaliphilic *Bacillus pseudofirmus* OF4 and *Bacillus halodurans* C-125 and plays a central role in the Na^+^ cycle, which is essential for the alkaline adaptation mechanisms (1, 2). In neutralophilic *Bacillus subtilis*, the Mrp complex acts as an Na^+^ and K^+^/H^+^ antiporter (2). Therefore, the 3 Mrp complexes of *Microbacterium* sp. strain TS-1 might also support the use of various cations as coupling ions of H^+^ influx for pH homeostasis.

The annotation also indicates that this bacterium has genes involved in chemotaxis/flagellar components, including one set of *mot* genes. Alkaliphilic *Bacillus pseudofirmus* OF4 and *Bacillus halodurans* C-125 have a *motPS* gene which encodes Na^+^-dependent flagellar motor stator proteins. In contrast, neutralophilic *Bacillus subtilis* has two sets of *mot* genes, the *motAB* gene, which encodes H^+^-dependent flagellar motor stator proteins, and the *motPS* gene (3). The amino acid residue that is important for H^+^ or Na^+^ selectivity has already been identified, and H^+^-dependent and Na^+^-dependent flagellar motor stator proteins can be determined (3). It is suggested that the *mot* genes of *Microbacterium* sp. strain TS-1 encode H^+^-dependent flagellar motor stator proteins. Therefore, it is proposed that *Microbacterium* sp. strain TS-1 has an H^+^-dependent flagellar motor and shows Na^+^-independent motility. It is unclear as to the mechanism whereby this bacterium uses the H^+^-dependent flagellar motor under alkaline conditions.

### Nucleotide sequence accession numbers.

The draft genome sequence of *Microbacterium* sp. strain TS-1 was deposited at DDBJ/EMBL/GenBank under the accession number BASQ00000000. The version described in this paper is the first version, BASQ01000000.
